# Neurotoxicity of HIV-1 Tat is attributed to its penetrating property

**DOI:** 10.1038/s41598-020-70950-x

**Published:** 2020-08-19

**Authors:** Xiaoli Tang, Huafei Lu, Bharat Ramratnam

**Affiliations:** Laboratory of Retrovirology, Division of Infectious Diseases, Department of Medicine, Rhode Island Hospital and Warren Alpert Medical School of Brown University, 55 Claverick Street (Laboratory 414), Providence, RI 02903 USA

**Keywords:** Biochemistry, Biological techniques, Biotechnology, Cell biology, Drug discovery, Microbiology, Molecular biology, Neuroscience, Diseases, Molecular medicine

## Abstract

We have recently engineered an exosomal Tat (Exo-Tat) which can activate latent HIV-1 in resting CD4+ T lymphocytes from antiretroviral treated HIV-1 infected patients. HIV-1 Tat protein can penetrate cell membrane freely and secrete into extracellular medium. Exo-Tat loses this penetrating property. HIV-1 Tat protein can damage the synaptic membranes contributing to the development of dementia in HIV-1 infected patients. To investigate whether the penetrating property attributes to synaptic damage in vivo, we have generated adeno-associated viruses AAV-Tat and AAV-Exo-Tat viruses. Vehicle control or AAV viruses (1 × 10^12 ^GC/mouse in 200 μl PBS) were injected into Balb/cj mice via tail veins. The mRNA and protein expression levels in blood, brain, heart, intestine, kidney, liver, lung, muscle and spleen were determined on day 21. Intravenously injected AAV-Tat or AAV-Exo-Tat mainly infects liver and heart. Short-term expression of Tat or Exo-Tat doesn’t change the expression levels of neuronal cytoskeletal marker β3-tubulin and synaptic marker postsynaptic density 95 protein (PSD-95). Wild-type Tat, but not Exo-Tat, reduces the expression level of synaptic marker synaptophysin significantly in mice, indicating that penetrating property of HIV-1 Tat protein attributes to synaptic damage.

## Introduction

Combination antiretroviral therapy (cART) has converted HIV-1 infection from a death sentence to a chronic disease. HIV-1 infected individuals can enjoy an almost normal lifespan if they adhere to the therapeutic regimen for life^1–3^. Since cART cannot eliminate HIV-1 virus from the body due to the existence of latent reservoirs, a “shock and kill” strategy was proposed to activate the latent HIV-1 viruses and the infected cells will die via cytopathic effect or are killed by immune system^4–6^. Several latency reversal agents (LRAs) were tested and failed in clinical trials due to incomplete activation of latent reservoirs^7,8^. HIV-1 Tat protein is one of the most potent LRAs^9,10^. The big concerns to use Tat as an LRA in clinic are the difficulty of delivering an antigenic protein and its neurotoxicity to central nervous system^11,12^. Chronic low-level expression of HIV-1 Tat protein in mice can damage the synapses demonstrating reduction of expression levels of neuroskeletal and/or synaptic markers such as the neuronal specific cytoskeletal protein βIII-Tubulin, the presynaptic vesicle protein p38 (Synaptophysin) and the postsynaptic density 95 protein (PSD-95)^13^. Mutational modification of Tat protein could attenuate its cytotoxicity and immunogenicity^12^. Recently, we have engineered an exosomal Tat which avoids its immunogenicity and can activate latent HIV-1 in primary, resting CD4+ lymphocytes from cART-treated HIV-1 infected patients^10^. Here we compare the short-term neurotoxicity of wild-type Tat and engineered Exo-Tat in Balb/cj mice, commonly used and readily available regular mice.

## Results

### Wild-type Tat, but not Exo-Tat, penetrates membrane

**Figure 1:**
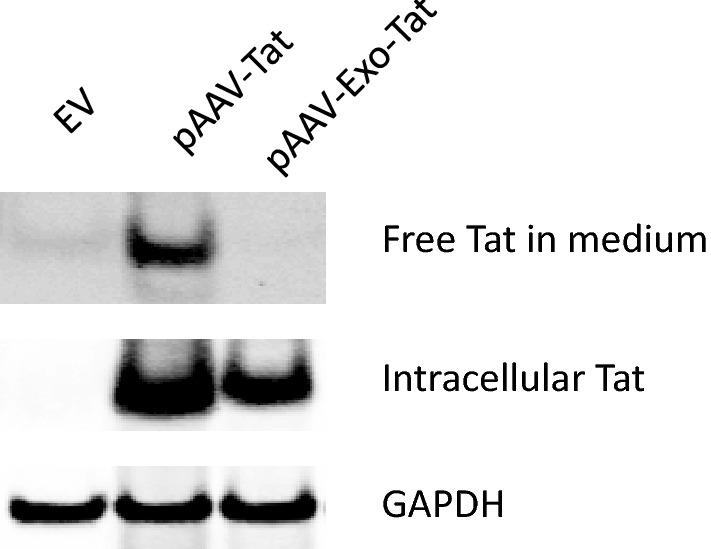
Expression of pAAV-Tat and pAAV-Exo-Tat in HEK293T cells. Empty vector (EV), pAAV-Exo-Tat or pAAV-Tat was transfected into 293 T cells by means of Lipofectamine 2000. Forty-eight hours posttransfection, the cells were harvested and seperated from the culture medium by centrifugation. The cell pellets were used for preparation of cell lysates. Twenty μg protein was used for western blot. The culture medium was collected and centrifuged at full speed for 10 min. The supernatants were transferred to clean tubes and 50 μl anti-HA mAb conjugated sepharose beads were added to each tube. After rotate at 4 °C overnight, the beads were washed in IP lysis buffer for three times. Thirty μl of 2XSDS loading buffer was added to each tube to elute the bound protein for western blot. GAPDH was used as a lysate loading control.

We have recently engineered an exosomal Tat (Exo-Tat) which can be loaded into exosomes. Exosomes loaded with HIV-1 Tat protein activate latent HIV-1 in primary, resting CD4+ T cells from cART-treated HIV-1-infected patients^10^. We reasoned if the cells in human body can generate exosomes containing Tat protein, these Tat exosomes may activate latent HIV-1 from the reservoirs of HIV-1 infected patients. Therefore, in a “shock and kill” therapeutic regimen, the HIV-1 infected cells will be cleared via cytopathic effect or by immune system. We first intend to test the possibility to generate Tat-loaded exosomes in mice. Since adeno-associated virus is the only non-pathogenic virus and already approved by FDA for clinical use^14^, we are going to take this advantage to utilize AAV to deliver exosomal Tat cDNA into mouse cells in vivo. We subcloned wild-type Tat and Exo-Tat into the expression vector pAAV-MCS resulting in pAAV-Tat and pAAV-Exo-Tat. To check Tat protein expression in vitro, empty vector pAAV-MCS (EV), wild-type Tat expression vector pAAV-Tat or exosomal Tat expression vector pAAV-Exo-Tat was transfected into HEK293T cells respectively. Both Tat and Exo-Tat expressed in cells very well. But only wild-type Tat could secrete into the culture medium as free Tat protein (Fig. 1) indicating wild-type Tat but not exosomal Tat penetrates cell membranes. In the supernatant of pAAV-Exo-Tat transfected HEK293T cells, Exo-Tat was only detected in the exosomes (data not shown).

### AAV virus production and in vitro infectivity

**Figure 2:**
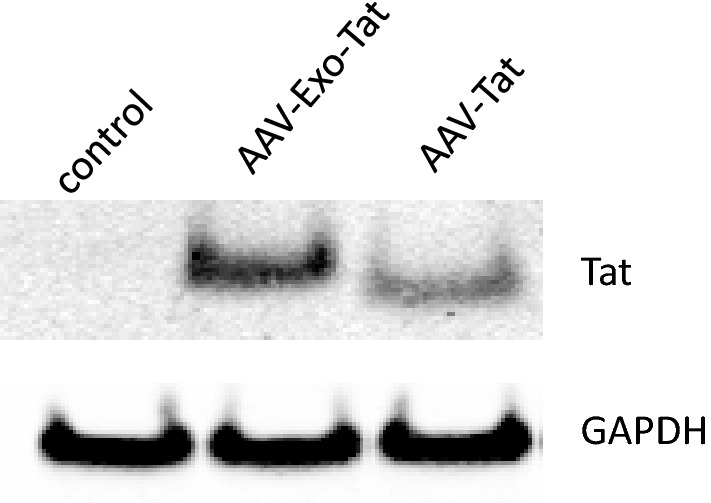
In vitro infectivity of Adeno-associated viruses. Vehicle control (EV), AAV-Exo-Tat (7.88 × 10^9 ^GC) or AAV-Tat (7.8 × 10^9 ^GC) was added into 1 × 10^6^ MOLT-4 cells. Forty-eight hours post-infection, the cells were harvested for preparation of cell lysates (25 μl). Twenty μl lysate was used for western blot. GAPDH was used as a lysate loading control.

To generate AAV-Tat or AAV-Exo-Tat viruses, pAAV-Tat or pAAV-Exo-Tat was co-transfected with vectors pAAV-DJ and pHelper at 1:1:1 ratio into HEK293T cells. Production of AAV viruses were conducted at a cGMP facility of Vigene Biosciences. The in vitro infectivity was tested using MOLT-4 cells. The result showed that these AAV viruses infected MOLT-4 cells very well in cell culture system (Fig. 2).

### In vivo infectivity of AAV-Tat and AAV-Exo-Tat

**Figure 3:**
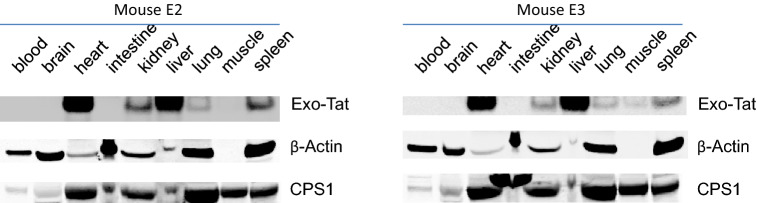
In vivo expression of Exo-Tat protein in mice. The cDNA sequence encoding Exo-Tat was subcloned into an adeno-associated virus vector pAAV-MCS. Subsequenced vector pAAVExo-Tat was co-transfected with pAAV-DJ and pHelper at 1:1:1 ratio into HEK293T cells to generate AAV-Exo-Tat viruses. AAV-Exo-Tat viruses (1 × 10^12 ^GC in 200 μl PBS) were injected into mice via tail vein. Three weeks later, the mice were anesthetized with isoflurane and blood was drawn by cardiac puncture. Brain, heart, intestine, kidney, liver, lung, muscle and spleen were taken immediately. Tissue lysates were prepared by homogenizing the tissue in IP lysis buffer (Pierce). Two hundred microgram of each tissue lysate was used to immuno-precipitate Exo-Tat. Precipitated proteins were eluted for western blot. Another 10 μg of each tissue lysate was used for western blot to measure β-Actin and CPS1 expression as loading controls. Note: Mice E2 and E3 were two mice injected with AAV-Exo-Tat viruses.

**Figure 4:**
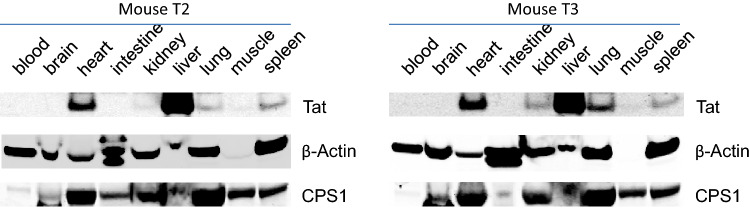
In vivo expression of HIV-1 Tat protein in mice. The cDNA sequence encoding HIV-1 Tat was subcloned into an adeno-associated virus vector pAAV-MCS. Subsequenced vector pAAV-Tat was co-transfected with pAAV-DJ and pHelper at 1:1:1 ratio into HEK293T cells to generate AAV-Tat viruses. AAV-Tat viruses (1 × 10^12 ^GC in 200 μl PBS) were injected into mice via tail vein. Three weeks later, the mice were anesthetized with isoflurane and blood was drawn by cardiac puncture. Brain, heart, intestine, kidney, liver, lung, muscle and spleen were taken immediately. Tissue lysates were prepared by homogenizing the tissue in IP lysis buffer (Pierce). Two hundred microgram of each tissue lysate was used to immuno-precipitate Tat. Precipitated proteins were eluted for western blot. Another 10 μg of each tissuelysate was used for western blot to measure β-Actin and CPS1 expression as loading controls. Note: Mice T2 and T3 were two mice injected with AAV-Tat viruses.

**Figure 5:**
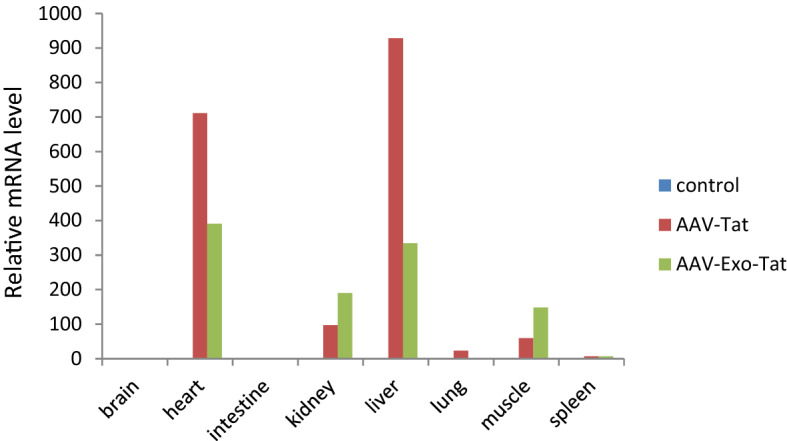
Tat or Exo-Tat mRNA levels in mouse organs. The cDNA sequence encoding Exo-Tat was subcloned into an adeno-associated virus vector pAAV-MCS. Subsequenced vector pAAV-Tat or pAAV-Exo-Tat was co-transfected with pAAV-DJ and pHelper at 1:1:1 ratio into HEK293T cells to generate AAV-Tat or AAV-Exo-Tat viruses. AAV viruses (1 × 10^12 ^GC in 200 μl PBS) were injected into mice via tail vein. Three weeks later, the mice were anesthetized with isoflurane and blood was drawn by cardiac puncture. Brain, heart, intestine, kidney, liver, lung, muscle and spleen were taken immediately. Total RNA was extracted from each organ using Trizol reagent. mRNA levels were measured by qRT-PCR. The mean relative copy number of 3 mice is shown here.

Vehicle control (200 μL PBS) or AAV virus (1 × 10^12 ^GC/mouse in 200 µl PBS) was injected into Balb/cj mice via tail veins. Twenty-one days after iv injection, blood was taken by cardiac puncture under isoflurane anesthesia. Heart, liver, spleen, kidney, lung, muscle, intestine and brain were taken out for extracting total RNA and proteins. Tat or Exo-Tat mRNA levels were determined by qRT-PCR and protein levels were measured by western blot. Western blot results showed that AAV-Tat and AAV-Exo-Tat viruses mainly infected mouse liver and heart (Figs. 3, 4). The qRT-PCR results were consistent with the western blot results (Fig. 5).

### Tat protein levels in exosomes and brain

**Figure 6:**
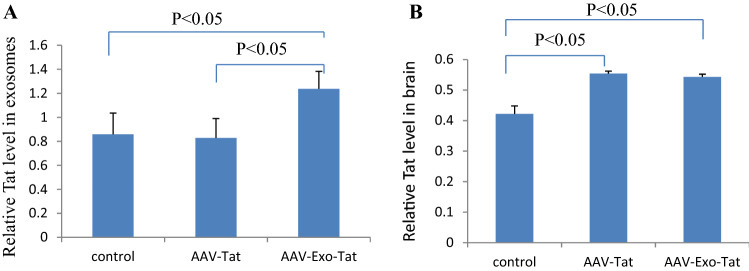
Relative Tat levels in exosomes or brain by ELISA. (**A**) Exosomes were isolated from mouse plasma by differential ultracentrifugation. IP lysis buffer (Pierce) was used to release proteins from exosomes. Tat levels were determined using a modified ELISA protocol. (**B**) Mouse brain was homogenized in IP lysis buffer to prepare brain lysate. One hundred μL was used for Tat ELISA analysis. Note: n = 3, *p* < 0.05 indicates statistically significant.

Our previous in vitro experiments showed that engineered Exo-Tat but not wild-type could express in exosomes. We wonder if mouse cells can generate Tat-loaded exosomes after iv injection of AAV viruses encoding Tat or Exo-Tat cDNA sequences. Since we just had limited mouse plasma (0.5 mL per mouse), we detected Tat expression in exosomes using more sensitive ELISA instead of western blot. Tat protein could be detected only in the exosomes of AAV-Exo-Tat infected mice (Fig. 6A). Although western blot failed to detect Tat protein expression in mouse brains of AAV-Tat or AAV-Exo-Tat infected mice (Figs. 3, 4), trace Tat level could be detected by ELISA (Fig. 6B).

### Wild-type Tat, but not Exo-Tat, induces synaptophysin reduction

**Figure 7:**
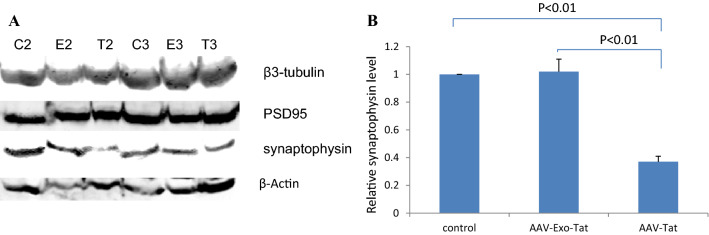
HIV-1 Tat protien, but not the modified Exo-Tat, induces reduction of synaptophysin in mouse brain. Vehicle control (200 μl PBS ), AAV-Exo-Tat viruses or AAV-Tat viruses (1 × 10^12 ^GC in 200 μl PBS) were injected into mice via tail vein. Three weeks later, the mice were anesthetized with isoflurane. The whole brain was taken out. About 500 μg of brain was used to prepare brain lysate. Twenty microgram of brain lysate was used for western blot to determine the expression levels of neuronal cytoskeletal maker β3-tubulin and synaptic markers postsynaptic density 95 protein (PSD95) and presynaptic vesicle protein p38 (synaptophysin). β-Actin was used as a loading control. (**A**) Protein expression levels by western blot (C2: control mouse 2, E2: AAV-Exo-Tat mouse 2, T2: AAV-Tat mouse 2, C3: control mouse 3, E3: AAV-Exo-Tat mouse 3, T3: AAV-Tat mouse 3). (**B**) Relative synaptophysin level normalized to β-actin level. Note: n = 3, *p* < 0.05 indicates statistically significant.

Neurotoxicity of HIV-1 Tat protein manifests synaptic damage as indicated by decreasing expression levels of neuronal cytoskeletal marker β3-Tubulin and synaptic markers presynaptic vesicle protein p38 (synaptophysin) and postsynaptic density 95 protein (PSD95). We wonder if the trace level Tat in mouse brain can damage mouse synapses. Our results showed that infection of mice for 21 days with AAV-Exo-Tat didn’t change the expression levels of β3-Tubulin, PSD95 or synaptophysin. Infection of mice with AAV-Tat for 21 days didn’t change the expression levels of β3-Tubulin or PSD95 but significantly reduced synaptophysin expression level (Fig. 7), indicating that neurotoxicity of HIV-1 Tat protein is attributed to its penetrating property.

## Discussion

Exosomes are virtually generated by all types of cells in the body and play very important roles in cell functions^15,16^. Proteins in exosomes evade the immune recognition^15^. After we have confirmed the latency reversal function of exosomal Tat in ex vivo experiments^10^, we wonder if we can transfer the cells in the body into exosomal Tat factories. Autologous exosomes loaded with Tat protein can evade the immunogenicity and the cleavage by proteases. Since AAV is the only nonpathogenic virus and is approved by FDA for clinical use^14^, we have constructed AAV viruses encoding HIV-1 Tat and engineered Exo-Tat, namely AAV-Tat and AAV-Exo-Tat. We produced these viruses at the cGMP facility of Vigene Biosciences using expression vector (pAAV-Tat or pAAV-Exo-Tat):pAAV-DJ:pHelper = 1:1:1 ratio recommended by the manufacturer of AAV-DJ Helper Free Expression System (Cell Biolabs). Unfortunately, the final titers of AAV viruses were much lower than expected, resulting in only 3 mice used in each group instead of planned 10 mice for each group. We are optimizing the production of AAV viruses.

After infection of mice with AAV-Tat or AAV-Exo-Tat for 21 days, Tat or Exo-Tat mRNA couldn’t be detected in mouse brains, indicating very few or no AAV viruses penetrated the blood brain barrier (BBB). The trace level of Tat protein in mouse brain must be from circulating free Tat which freely penetrates the BBB. The trace level of Exo-Tat protein in mouse brain should be from exosomes containing Exo-Tat considering exosomes can enter nervous system^17^. Since Tat or Exo-Tat protein level was below the detection limit of western blot, Tat ELISA assay was used to detect the trace protein levels. All the available commercial Tat ELISA kits measure the Tat antibody level but not the antigen level. We modified a Tat ELISA protocol using various reagents from multiple vendors. The primary antibody detected multiple nonspecific bands in addition to the specific band, resulting in a high background level in the control group. Further modification of the Tat ELISA protocol is underway.

The mechanisms of HIV-1 cytotoxicity have been controversial^11,18–20^. The Tat cytotoxicity in cell culture system is pretty low. Intracranial injection may complicate the interpretation by local damage. Transgenic mouse models never got consistent results, one may induce tumors and another may lead dementia depending the insertion sites of Tat gene into genomic DNA. We hypothesized that penetrating property of HIV-1 Tat attributes to its neurotoxicity by damaging membranes. The early and sensitive indicator of synaptic damage is the reduction of synaptic markers such as synaptophysin and PSD95. We engineered an exosomal Tat which loses the penetrating property. Expression of HIV-1 Tat for 21 days reduces synaptophysin level in mouse brain. Expression of Exo-Tat doesn’t change the levels of neuronal cytoskeletal or synaptic markers, confirming our hypothesis. We will further confirm this hypothesis using humanized HIV-1 latency model mice for long term study.

## Methods

### Cell culture and transfection

Cell culture and transfection were performed as previously described^10^. Briefly, HEK293T cells were cultured in Dulbecco’s modified Eagle’s medium (Life Technologies) with 10% fetal bovine serum (FBS) (Thermo Scientific), 2 mM l-glutamine and non-essential amino acids (Life Technologies). MOLT-4 cells were cultured in RPMI Medium 1,640 (Life Technologies) with 10% FBS. HEK293T cells were transfected with Lipofectamine when cell confluency was ~ 70%. MOLT-4 cells were transfected with Lipofectamine LTX Plus Reagent (Life Technologies)^10^.

### Construction of expression vectors

Exo-Tat is a recently engineered exosomal protein which a membrane associated peptide was added to the N-terminus of Tat and a nuclear localization signal was added to its C-terminus^10^. cDNA sequences encoding Tat or Exo-Tat were subcloned into an expression vector pAAV-MCS (System Biosciences) between EcoRI and BglII sites resulting in expression vector pAAV-Tat or pAAV-Exo-Tat. For convenience of detecting Tat or Exo-Tat protein, an HA-tag was added to the C-terminal end of Tat or Exo-Tat. The expression vectors were sequenced at Yale Keck Sequencing Facility.

### Production of AAV viruses

AAV-Tat or AAV-Exo-Tat viruses were generated by co-transfection the expression vector pAAV-Tat or pAAV-Exo-Tat with pAAV-DJ and pHelper (System Biosciences) at 1:1:1 ratio into HEK293T cells. All the AAV viruses were produced at the cGMP facility of Vigene Biosciences. The titers of AAV-Tat were 3.9 × 10^12 ^GC/mL and 6.95 × 10^12 ^GC/mL. The titers of AAV-Exo-Tat were 2.17 × 10^12 ^GC/mL and 1.97 × 10^12 ^GC/mL. Each vial of AAV was in 250 μL PBS.

### Animals

The experimental protocol was approved by the Lifespan Animal Care and Use Committee. The approval number is 505517. All experimental procedures were conducted in accordance with guidelines for the ethical treatment of animals. Balb/cj mice (3 months old) were purchased from Jackson Laboratory. Vehicle control or AAV viruses were injected into mice via tail veins (n = 3, 1 × 10^12 ^GC/mouse in 200 μl PBS). The mice were kept in a BL2 animal facility at Rhode Island Hospital and taken care by a certified technician. The mice were euthanized on day 21 by overdose isoflurane. Blood was taken by cardiac puncture before death. Brain, heart, intestine, kidney, liver, lung, muscle and spleen were taken after death.

### Semiquantitative RT-PCR

Total RNA was extracted from mouse organs with TRIZOL reagent (Invitrogen) and 1 μg of total RNA was used for cDNA synthesis using MMLV reverse transcriptase (New England Biolabs) as described in the manufacturer’s manual. Two μl of cDNA was used for PCR reaction to check Tat or Exo-Tat expression. GAPDH was used as control for normalization.

### Western blotting (WB)

WB was performed as previously described^21^. Briefly, cell or tissue lysates were separated by 10% SDS–PAGE electrophoresis and electroblotted to nitrocellulose membrane (Bio-Rad). Blotted membranes were probed with their respective primary antibodies, rotating at 4 °C overnight. Membranes were washed three times in TBST buffer and probed with secondary antibody (IRDye800-conjugated Affinity Purified IgG) at room temperature for 1 h. Membranes were then washed three times in TBST buffer and direct infrared fluorescence detection was performed with a Licor Odyssey Infrared Imaging System^21^.

### Tat ELISA

Since we couldn’t find a good commercial Tat ELISA kit. We used a modified Tat ELISA protocol. Briefly, 96-well plate (USA Scientific) was coated with 0.2 μg HA-tag rabbit monoclonal antibody (Cell Signaling) per well. Wash the wells with TBST and block the wells with blocking buffer (Licor) for 1 h at room temperature. Add 100 μL sample to specific wells and incubate at 4 °C overnight. After washing with TBST, Anti-HIV1 tat antibody (Biotin) (Abcam) in blocking buffer with 1:100 dilution was added to each well and incubate at room temperature for 2 h. Wash the wells with TBST for three times. Incubate with 100 μl diluted Streptavidin-HRP (1:100 dilution) (PerkinElmer) for 30 min at room temperature. After washing with TBST for 3 times, incubate with 100 μl OPD (O-Phenylenediamine Dihydrochloride) (PerkinElmer) for 30 min at room temperature, add 100 μl stop solution (PerkinElmer) to terminate the reaction, measure the absorbance at wavelength 490 nm using SpectraMax M5.

### Statistical analysis

Quantitative data were analyzed by unpaired Student’s *t* test to compare two groups. Data are expressed as mean ± standard error of mean. A *p* value < 0.05 indicates statistical significance.

## Supplementary information

Supplementary Information.
