# Supplementary material for: Neurotoxicity of HIV-1 Tat is attributed to its penetrating property
Source: Sci Rep. 2020 Aug 19;10:14002. doi: 10.1038/s41598-020-70950-x (PMC7438513; doi:10.1038/s41598-020-70950-x)
Supplement: Supplementary file 1 — Supplementary Information. [file 41598_2020_70950_MOESM1_ESM.pptx]

## Slide 1
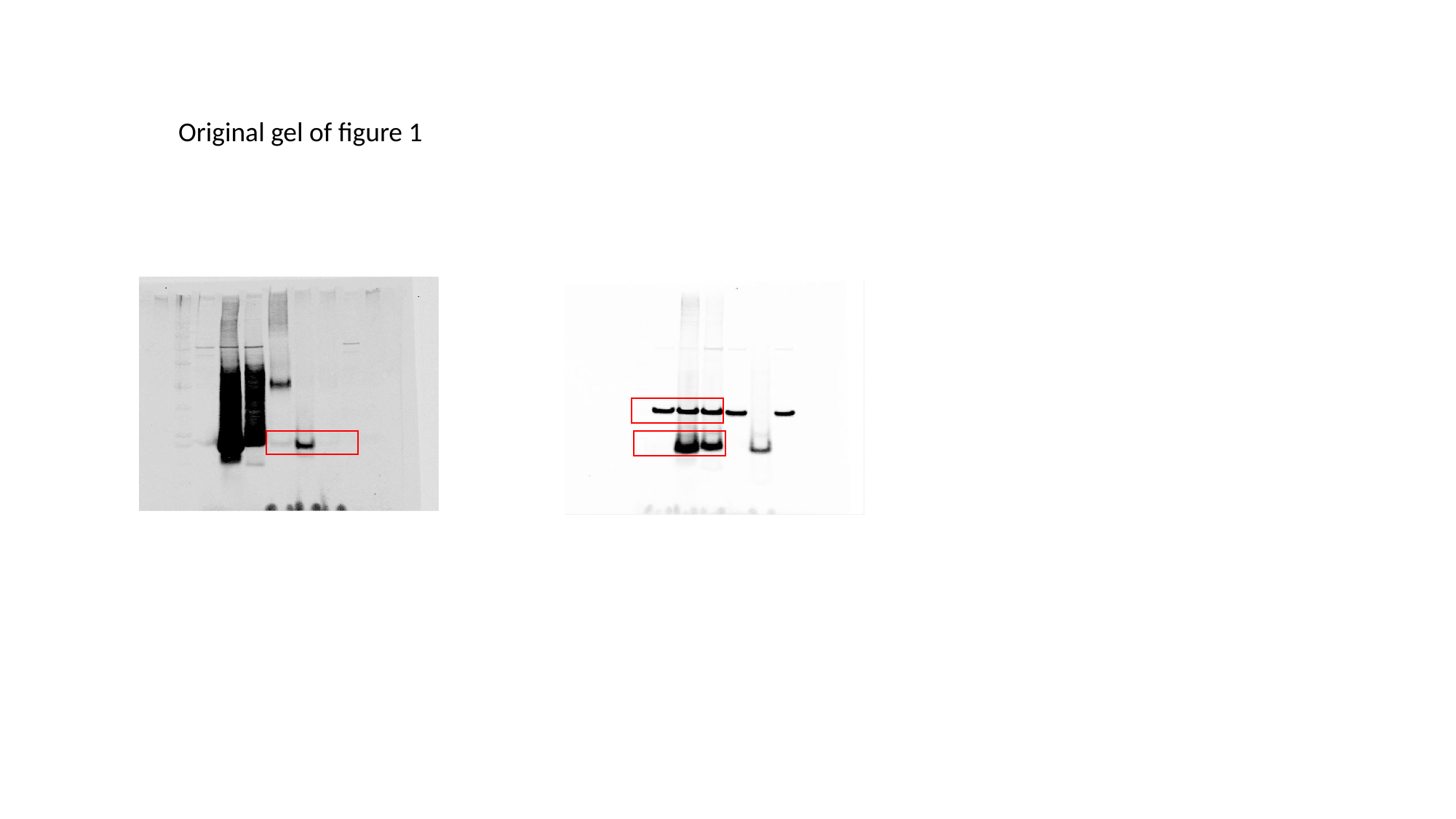

Original gel of figure 1

## Slide 2
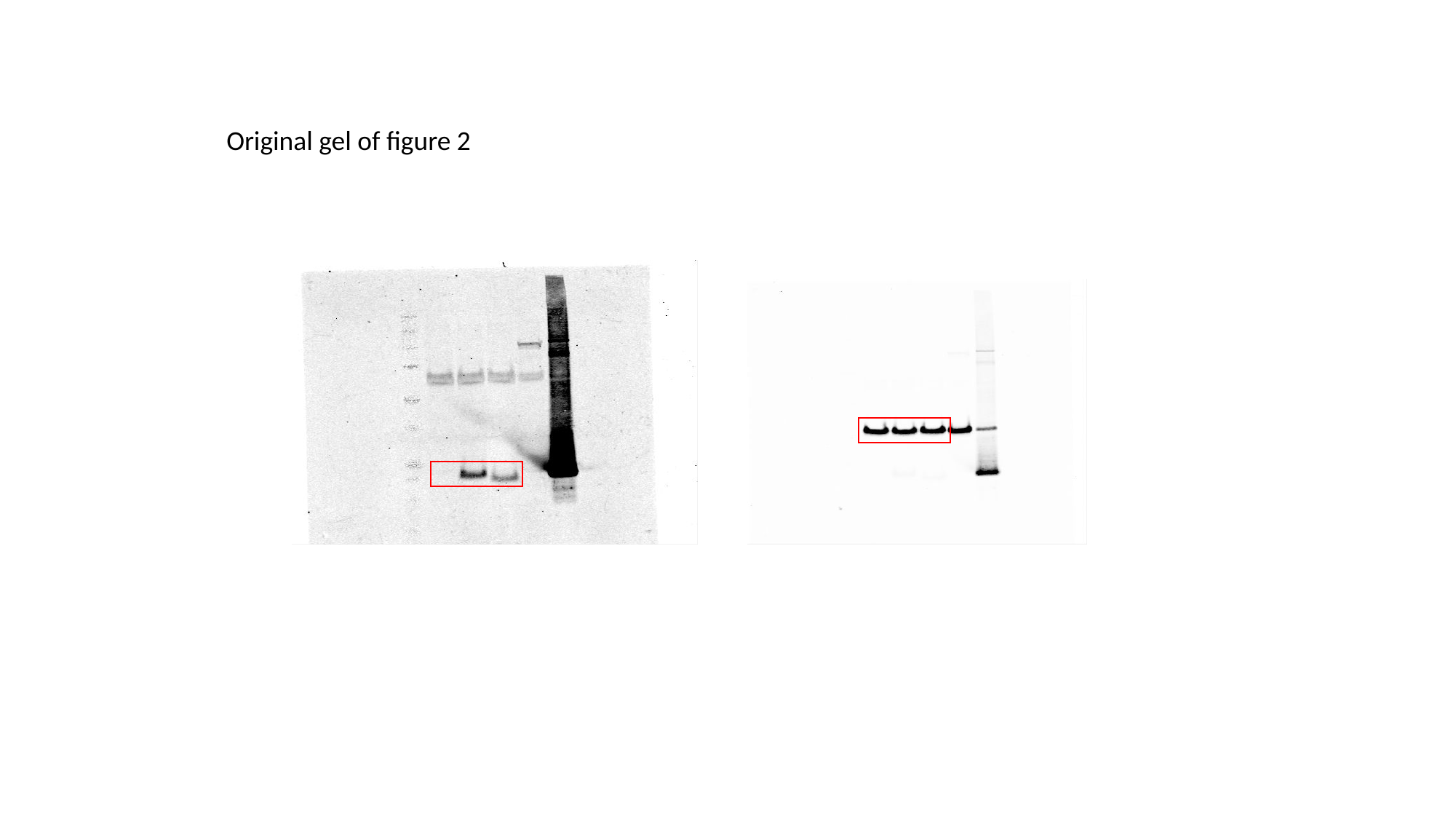

Original gel of figure 2

## Slide 3
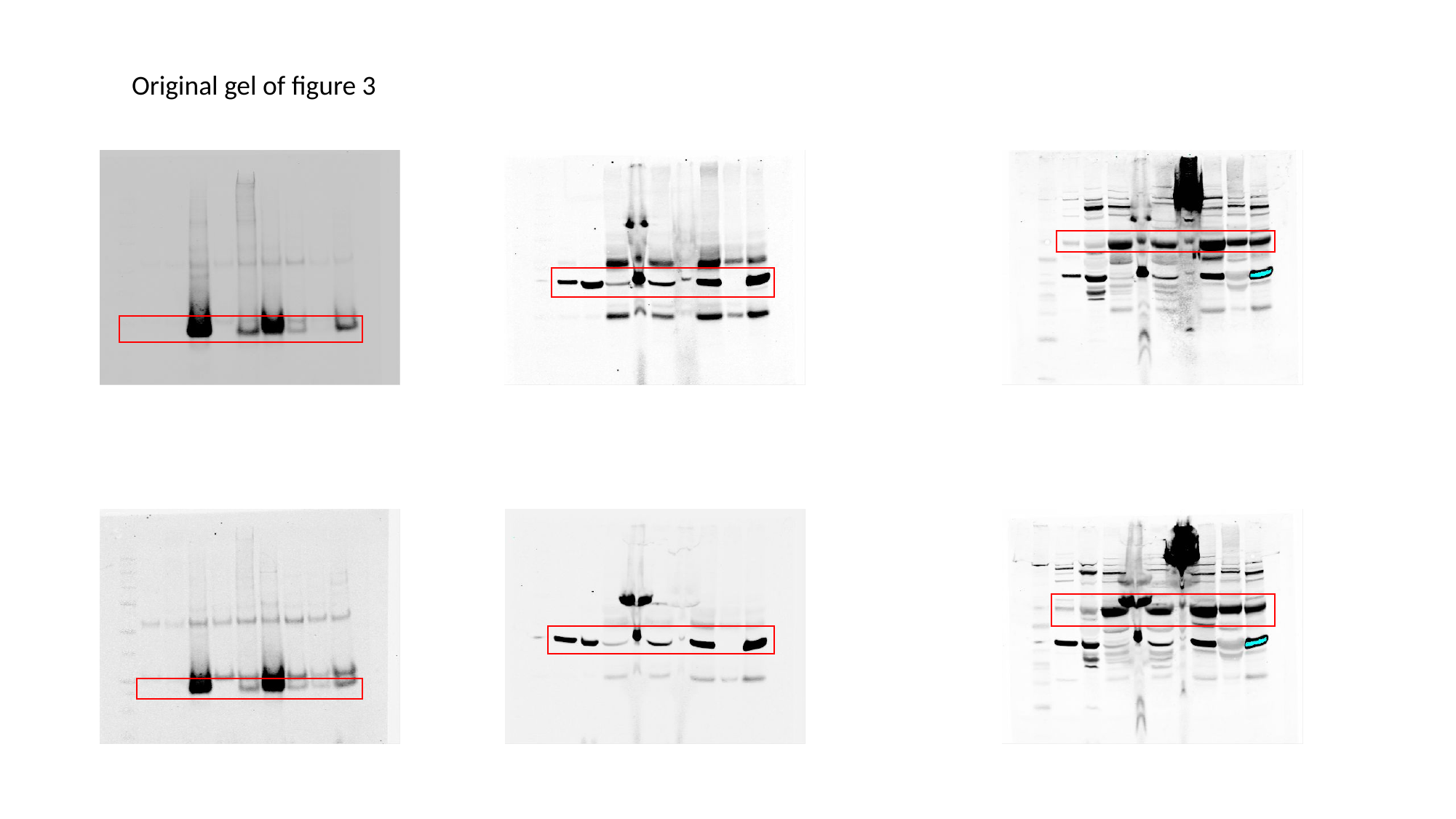

Original gel of figure 3

## Slide 4
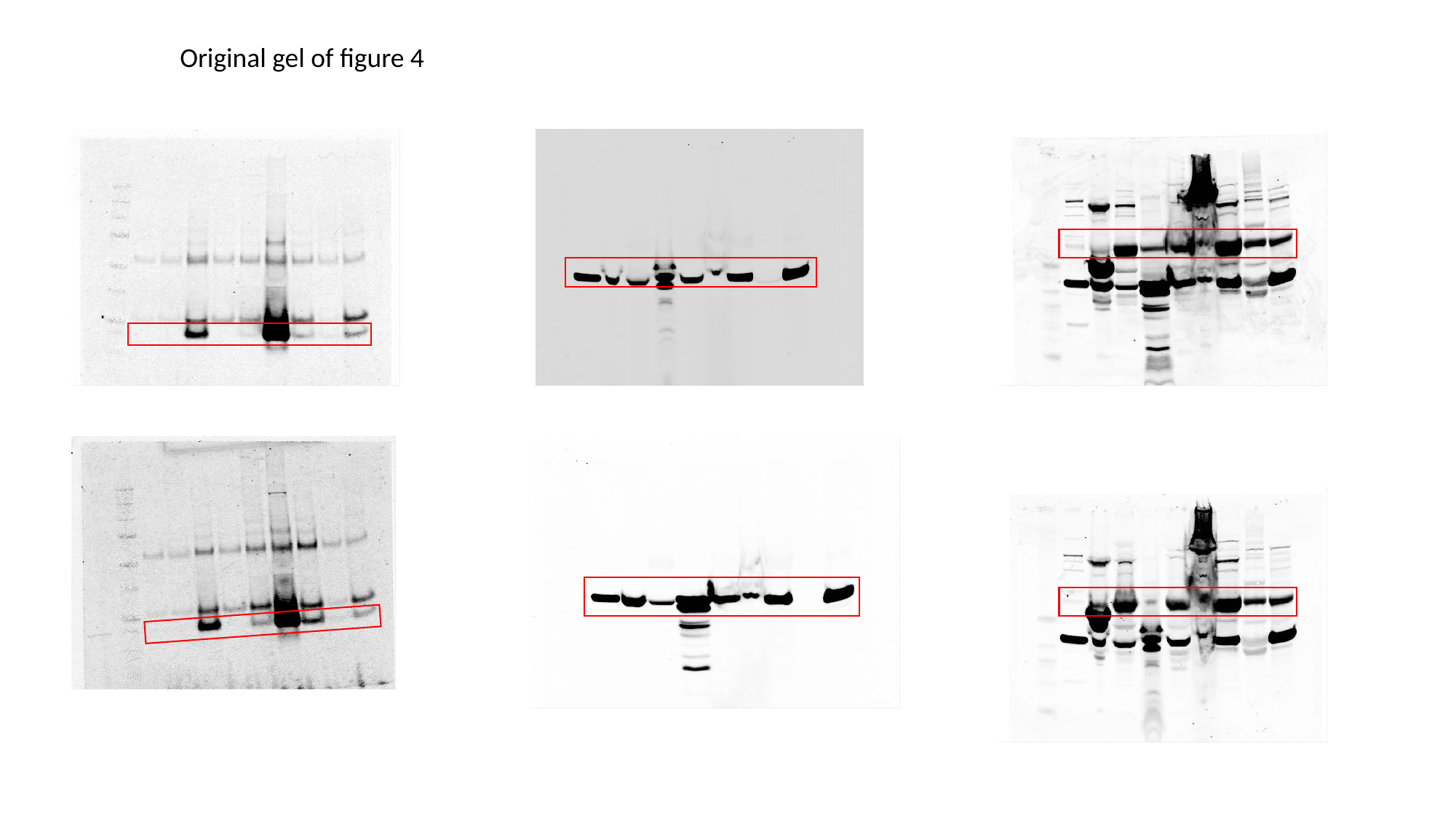

Original gel of figure 4

## Slide 5
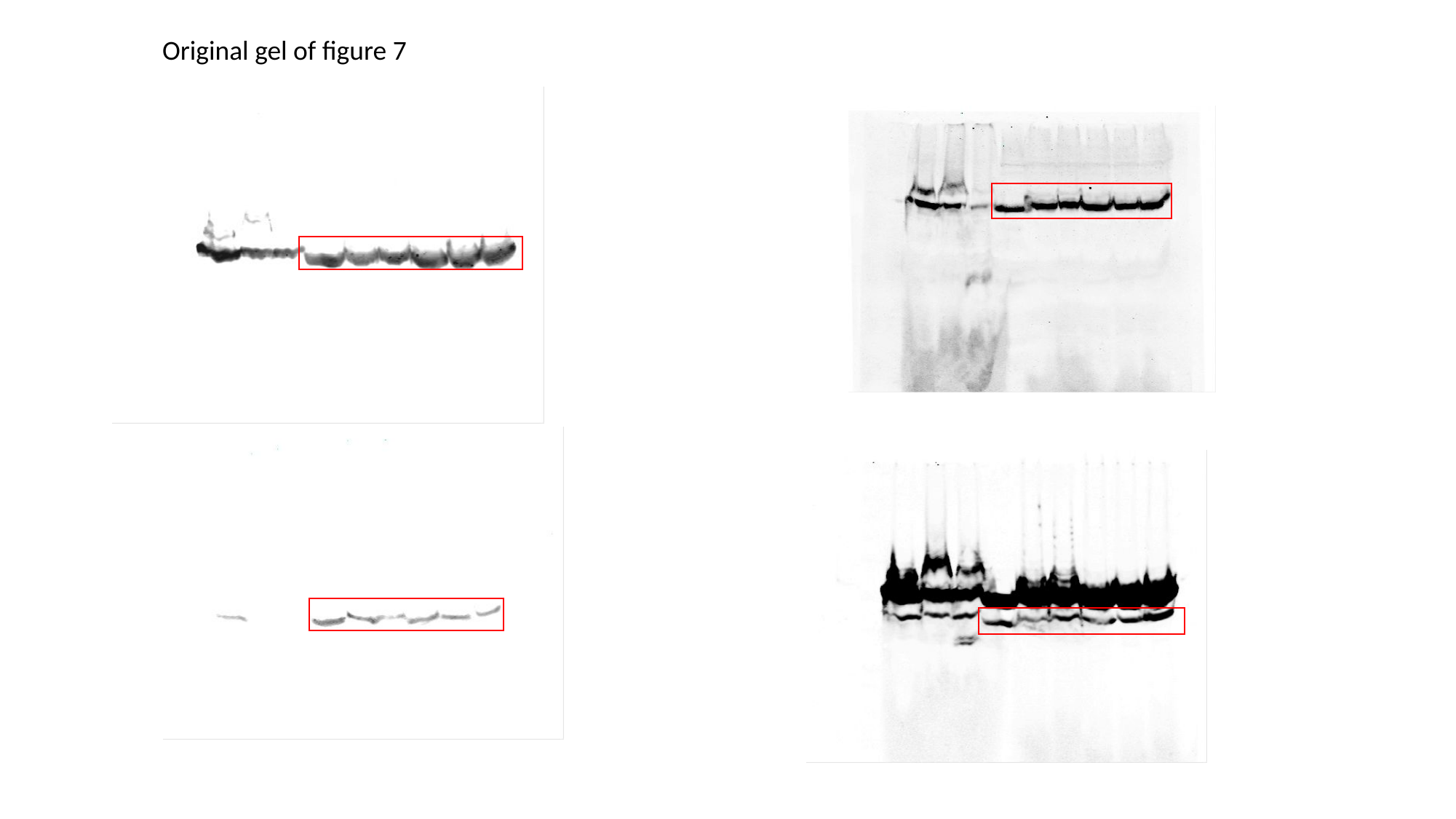

Original gel of figure 7
